# Adaptive Optics-Assisted Identification of Preferential Erythrocyte Aggregate Pathways in the Human Retinal Microvasculature

**DOI:** 10.1371/journal.pone.0089679

**Published:** 2014-02-26

**Authors:** Shigeta Arichika, Akihito Uji, Sotaro Ooto, Kazuaki Miyamoto, Nagahisa Yoshimura

**Affiliations:** Department of Ophthalmology and Visual Sciences, Kyoto University Graduate School of Medicine, Kyoto, Japan; National Eye Institute, United States of America

## Abstract

**Purpose:**

To characterize human parafoveal blood flow using adaptive optics scanning laser ophthalmoscopy (AO-SLO).

**Methods:**

In 5 normal subjects, erythrocyte aggregate distributions were analyzed on 3 different days. Erythrocyte aggregates were described as a “dark tail” in AO-SLO. The characteristics of the pathways with dark tail flow in the parafovea were measured. Additionally, the tendency for dark tail flow before and after bifurcations was analyzed to study the blood flow in detail.

**Results:**

Average velocity in parent vessels with dark tail flow was 1.30±0.27 mm/s. Average velocity in daughter vessels with dark tail flow was 1.12±0.25 mm/s, and the average velocity of plasma gaps in daughter vessels without dark tail flow was 0.64±0.11 mm/s. Downstream from the bifurcations, the velocity in vessels with dark tail flow was higher than that in those without it (p<0.001), and the branching angles of vessels with dark tail flow were smaller than those of vessels without it (p<0.001).

**Conclusions:**

Images from the AO-SLO noninvasively revealed pathways with and without dark tail flow in the human parafovea. Pathways with dark tail flow in the daughter vessels generally had faster flow and smaller bifurcation angles than daughter vessels without dark tail flow. Thus, AO-SLO is an instructive tool for analyzing retinal microcirculatory hemodynamics.

## Introduction

Ophthalmoscopy is a standard procedure in every routine eye examination and is useful in detecting retinal changes caused directly by eye disease and those secondary to systemic disease. This includes retinal blood vessel changes resulting from high blood pressure [1], [2], [3], arteriosclerosis [4], and diabetes mellitus. [5] Detecting the earliest visible signs of diabetic retinopathy (e.g., microaneurysms and dot hemorrhages) or staging systemic hypertensive and arteriosclerotic changes (using the Keith-Wagener [3] and Scheie [4] classification systems) are important in preventing systemic disease processes.

In addition to ophthalmoscopic examination, other approaches have been used to analyze the retinal circulation in more detail. Fluorescein angiography (FA) has long been the prevalent technique and the gold standard for evaluating retinal circulation, even with the potential side effects of fluorescein and the discomfort associated with the procedure. These drawbacks may prevent normal subjects and patients with early disease from undergoing angiography. Therefore, other tools that do not rely on an angiographic agent for measuring retinal blood flow have been developed, including laser Doppler velocimetry [6], scanning laser Doppler flowmetry [7], laser speckle flowmetry [8], and retinal functional imager. [9] Additionally, improved optical coherence tomography (OCT) systems, such as the Doppler OCT [10], can be used to examine retinal hemodynamics without a contrast agent.

Nishiwaki et al. [11], [12], Miyamoto et al. [13], and Kimura et al. [14] evaluated the animal retinal microcirculation using acridine orange and a scanning laser ophthalmoscope (SLO) system. Interestingly, they reported that leukocytes, when faced with a bifurcation, preferentially distribute themselves into the branch with the higher flow rate. [12] They concluded that these preferential pathways had a lower resistance and protected the retina by preventing leukocytes from entering small capillaries, where they would likely plug the small vessels. Unfortunately, acridine orange is toxic and cannot be used to study this phenomenon in humans.

Adaptive optics (AO) ophthalmoscopy was recently developed and has the ability to visualize photoreceptors [15], [16] and evaluate the retinal nerve fiber bundle [17] retinal blood flow [18], [19], [20], blood corpuscles [21], [22], and retinal vasculature [23] Cells visualized with adaptive optics scanning laser ophthalmoscopy (AO-SLO) included leukocytes, erythrocyte aggregates, and plasma gaps, and the visualization was achieved with a noninvasive, objective, agent-free approach. Specifically, AO-SLO [18], [19], [24], [25], [26], [27] was used to measure blood velocity in larger vessels [27] and leukocyte velocity in the parafovea. [19], [28] It was also used to noninvasively characterize plasma gaps and single-file flow of leukocytes [18] in humans. We previously reported bright particles moving in capillaries, which we suspect may be reflections of the photoreceptor, visible when circulating transparent objects (e.g., leukocytes or plasma gaps) pass over the photoreceptor. We also described the “dark tail,” which we interpreted as a region darker than the vessel shadow, that may correspond to aggregated erythrocytes upstream of leukocytes. Interestingly, the dark tail elongated in a time-dependent manner. [22] Erythrocyte aggregates are considered to be an important hemorheological determinant of microcirculatory problems [29], [30], [31] and an important hemorheological parameter because of their direct effects on whole blood viscosity. [32] Thus, direct monitoring of retinal erythrocyte aggregates in patients may provide early indications not visible on ophthalmoscopy of a microcirculatory disorder.

Here, we use the AO-SLO to examine erythrocyte aggregate (dark tail) distribution in the retinal capillary network in humans. We also analyze flow preferences of these dark tails in the parafovea.

## Methods

This study was approved by the Institutional Review Board and the Ethics Committee at Kyoto University Graduate School of Medicine. The study conduct adhered to the tenets of the Declaration of Helsinki. Written informed consent was obtained from each participant after the nature of the study and the risks and benefits of study participation were thoroughly explained.

### Subjects

Videos of the parafoveal areas were acquired using AO-SLO in 5 healthy Japanese subjects. All subjects had a medical history free of ocular and systemic disease. The eyes of all subjects were dilated before AO-SLO image acquisition with 1 drop each of tropicamide (0.5%) and phenylephrine hydrochloride (0.5%). Following dilation, subjects were examined for approximately 20 minutes per eye in a seated posture. All subjects had normal ophthalmoscopic findings, blood pressure, and intraocular pressure (IOP). Subject B in this paper also served as subject A in a previous report. [22] All AO-SLO images were acquired specifically for this study. All subjects underwent AO-SLO imaging three times, with each examination separated by an interval of at least one week. This was done to ensure that dark tail measurements were reproducible in terms of distribution and flow direction. Each vessel was imaged for at least 30 seconds.

### Adaptive Optics Scanning Laser Ophthalmoscope Imaging

We developed a novel AO-SLO system (Canon Inc., Tokyo, Japan) with a high wavefront correction efficiency by using a dual liquid-crystal phase modulator (LCOS-SLM; X10468-02, Hamamatsu, Japan). [21], [22] The AO-SLO videos were acquired at 32 or 64 frames/s in the area covering the parafovea. The scan area was 2.8°×2.8° or 1.4°×2.8° at the retina and had a sampling of 400×400 or 200×400 pixels, respectively. Velocities of moving objects in vessel shadows were analyzed at a rate of 64 frames/s. All AO-SLO imaging procedures were performed with the optical focus on the photoreceptor layer. [22].

### Detection and Measurement of Dark Tail

**Figure 1:**
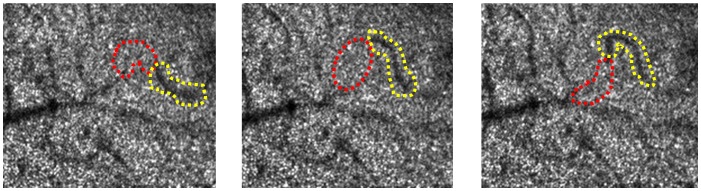
Consecutive AO-SLO images from a normal subject. Bright particles and dark regions were identified in vessel shadows on the cone mosaic. Bright particles are thought to represent leukocytes and plasma gaps. Dark regions represent erythrocyte aggregates, described as a “dark tail.” The red dot circles and yellow dot circles highlight bright particles and dark tails, respectively. Blood flow direction was from the right to the left side.

We investigated the shadows of aggregated erythrocytes that block the AO-SLO laser, creating a “dark tail.” The dark tail was defined as a dark region (darker than the vessel shadow) that occurred closely behind a bright, moving particle within a vessel (Figure 1).

**Figure 2:**
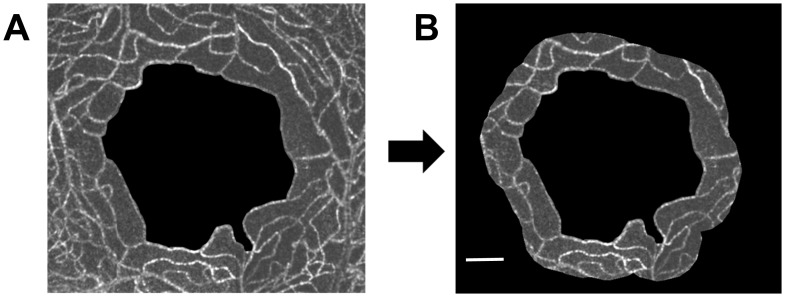
Extraction of parafoveal network data. (A) Montage of the parafoveal capillary network. The center black region represents the foveal avascular zone (FAZ). (B) Doughnut-shaped region within 150 µm of the FAZ edge. Vessel lengths were measured within the doughnut-shaped region and on the border of the FAZ. Scale bar represents 150 µm.

A montage of the parafoveal capillary network was manually created by overlapping constructed capillary projections (Figure 2A). Capillary images were constructed as projections of moving objects in sequential frames by using the motion contrast-enhancement technique [18], [25], which utilizes the AO-SLO Retinal Image Analyzer (ARIA, Canon Inc., Tokyo, Japan). [22] From these sequential frames, the foveal avascular zone (FAZ) boundary was manually determined. The region of interest (ROI) was automatically generated based on the FAZ boundary, according to the methods described by Tam et al. [25] The distance transform was then used to calculate how far pixels were outside of the FAZ boundary. Pixels inside of the FAZ and further than 150 µm from the edge of the FAZ were excluded from analyses (Figure 2B). This annulus surrounding the FAZ was regarded as a capillary monolayer in the human macula. [25].

**Figure 3:**
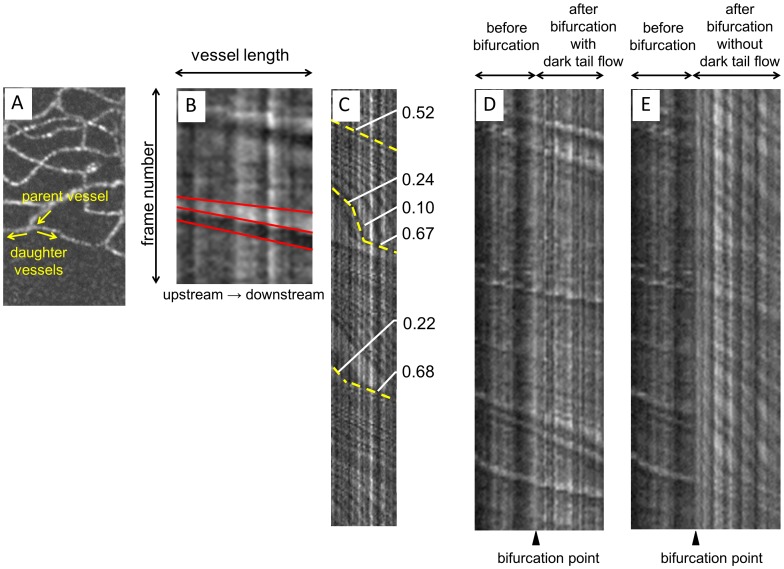
Interpretation and characteristics of spatiotemporal (ST) images. (A) Vessels before bifurcation were defined as parent vessels. Vessels after bifurcation were defined as daughter vessels. (B) Interpretation of ST images. Vessels with a dark tail, a white band, and a dark band were observed. The narrow white band corresponded to trajectories of bright moving objects, and the wide black band corresponded to trajectories of the dark tail. Dark tail velocity was calculated as the slope of the red line placed halfway between white and dark bands. As velocity decreased, the slope steepened. (C) An ST image of a vessel without a dark tail. Curved velocity changes were observed. Unlike vessels with a dark tail, velocities were not straight lines and seemed to change periodically (velocity varied between 0.10 and 0.68 mm/s over 2 s in this ST image). Numbers on the right side are yellow slope velocities showing changes every moment. (D) Combined ST images of dark tail vessels before and after bifurcation. (E) Combined ST images of a dark tail parent vessel and a non-dark tail daughter vessel. Sudden velocity decreases were observed at bifurcations.

In order to analyze dark tail distributions, we separated blood flow within the analytic region into pathways with dark tail flow and pathways without dark tail flow. The distinction between dark tail and non-dark tail flow was made through careful observation and analysis of AO-SLO movies and corresponding spatiotemporal (ST) images (Figure 3B). The length of the whole pathway was manually measured in both types of pathways, as well as the length along the FAZ boundary.

### Characterization of Blood Flow and Vessel Patterns at Bifurcations

Bifurcations consisting of a parent vessel with a dark tail flow, a daughter vessel with a dark tail flow, and a daughter vessel without a dark tail flow were chosen to analyze factors determining dark tail preferential pathways. Blood components were identified using ST images, according to the methods described by Tam et al. [18] Briefly, leukocyte traces were identified as (i) thick, (ii) high contrast, (iii) sparse, or (iv) unidirectional (Figure 3B). Plasma gap traces, which tended to have lower contrast than leukocyte traces, were identified as (i) thin or (ii) dense (Figure 3C). Dark tail traces following leukocyte traces were identified as (i) thick, (ii) high contrast, or (iii) hyporeflective (Figure 3B). [22].

#### Blood flow velocity measurement at bifurcations

All bifurcation images with sufficient quality were used to detect moving blood components, including dark tails, leukocytes, and plasma. At bifurcations, the dark tail flow in the parent vessel was bisected into dark tail flow and dark tail-free flow in the daughter vessels. The difference in blood flow velocity between the daughter vessel with and without a dark tail flow was determined by comparing dark tail velocity in the dark tail daughter vessel and plasma velocity in the non-dark tail daughter vessel. This method has been described in full in another study. [22] Briefly, sequential frames of the same vessel were compared, placing the length of the line on the horizontal axis and the frame number on the vertical axis. A white band, black band, and white line corresponded to the trajectories of moving leukocytes, dark tails (Figure 3B), and plasma gaps (Figure 3C), respectively. Dark tail and plasma gap velocities were obtained by calculating the reciprocal of the slope of the borderline between the white and black bands and the white line depicted in the ST image. In order to synchronize the velocity and the cardiac cycle, a pulse oximeter (Oxypal Neo, NIHON KOHDEN, Japan) was attached to the subject’s earlobe. As was done by Martin et al. [19], measurements were divided into five equal bins, each corresponding to the segment of the cardiac cycle in which they were observed. [19] To correct for the influence of the cardiac cycle on measured velocities, the average velocity during each of the 5 cardiac cycle segments was calculated separately. The total average velocity was then calculated by averaging each cycle segment velocity.

#### Measurement of capillary diameter

The diameters of 3 capillaries (1 parent and 2 daughter vessels) were measured at each bifurcation by using the constructed capillary images. [22] Values were then analyzed to determine the influence of vessel diameter on dark tail pathway preference. For each vessel, average diameter was manually calculated by taking measurements 10, 20, and 30 µm from the bifurcation. For each subject, diameters of 4 bifurcations were analyzed, yielding measurements for 20 bifurcations and 60 vessels.

#### Measuring angles between parent and daughter vessels

Angles between parent and daughter vessels were measured to determine the influence of angles on dark tail pathway preference. Measurements were made with the assistance of public-domain image analysis software (ImageJ, National Institutes of Health, Bethesda, MD). The standard line for measuring angles was drawn by joining the midpoints of the diameter of the parent vessel, which were determined at two points–at the center of the bifurcation and at a point 50 µm upstream of the bifurcation. Next, straight lines were drawn on the 2 daughter vessels in the same fashion. Lastly, angles between the extended parent vessel and each daughter vessel were measured. For each subject, 4 bifurcations were analyzed, yielding a study total of 20 bifurcations and 40 angles.

### Statistical Analyses

All values are presented as mean ± standard deviation (SD). Paired *t*-tests were used to examine the statistical significance of differences between dark tail and non-dark tail pathway length in the region 150 µm away from the FAZ, dark tail and non-dark tail pathway separations on the FAZ boundary, and the angle between daughter vessels in bifurcations. Comparisons of velocities and vessel diameters of the 3 vessels (1 parent and 2 daughter vessels) were carried out using repeated measures analysis of variance, and differences between the 2 groups were analyzed using the paired *t*-test, followed by Bonferroni correction**.** All calculations were performed by using StatView (ver. 5.0, SAS Inc., Cary, NC). A p value <0.05 was considered statistically significant.

## Results

### Distribution of Pathways Preferential to Dark Tails

**Figure 4:**
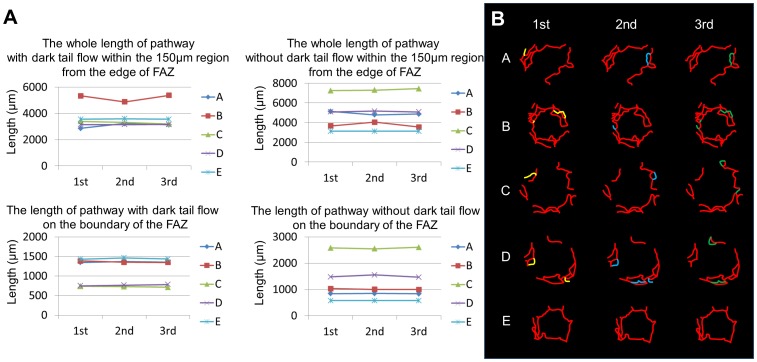
Daily variance of dark tail flow distribution. (A) Changes in the lengths of pathways with or without dark tail on three different days. Note that the lengths show little daily variance. Alphabet A, B, C, D, and E represent each subject. (B) Blood flow distributions of dark tails in the parafovea were nearly identical on each of 3 different days, with only minimal changes in 4 of 5 subjects. Red lines represent the same distribution of dark tails on the 3 different days. The yellow, blue, and green lines represent differences in distribution between days.

Mean subject age was 33.4±7.1 years (range, 23–41 years) and mean axial length was 24.8±1.2 mm (range, 23.6–26.5). In all 5 subjects, vessels could be divided into 2 groups based on the presence or absence of dark tail flow. The average length of dark tail vessels for 3 days was 3077±191 µm, and the average length of non-dark tail vessels was 4926±180 µm in Subject A. These numbers varied between subjects and measured 5192±272 and 3755±263 µm in Subject B; 3301±99 and 7327±106 µm in Subject C; 3150±11 and 5110±53 µm in Subject D; and 3566±17 and 3118±2 µm in Subject E, for dark tail and non-dark tail vessels, respectively (Figure 4A). The average lengths of dark tail vessels and non-dark tail vessels at the FAZ boundary, respectively, were 1360±12 and 844±5 µm in Subject A; 1362±20 and 1014±20 µm in Subject B; 727±12 and 2579±29 µm in Subject C; 766±16 and 1500±47 µm in Subject D; and 1444±17 and 576±2 µm in Subject E (Figure 4A). No significant pathway differences were found between dark tail flow and non-dark tail flow vessel groups in the measured region 150 µm away from the FAZ (p = 0.29) or at the FAZ boundary (p = 0.75).

### Variation in Dark Tail Flow Distribution

On the 3 different days that AO-SLO images were collected, only minimal variation in dark tail flow distribution was observed between measurements in 4 subjects (Figure 4B). In Subject A, distribution changes were found in 2 vessels among 3 measurements (from 1 branch to another). These distribution changes were also found in 3 vessels in Subject B and in 4 vessels each in Subjects C and D. In subject E, no changes were observed. Changes in the direction of dark tail flow were not observed in any subject.

### Differences between Pathways with and without Dark Tail Flow on Spatiotemporal Images

On pathways with a dark tail flow, a narrow white band and a wide black band, which represented a bright moving object’s and a dark tail’s trajectory, respectively, were observed on ST images. The trajectories of both bands were straight lines. On pathways without a dark tail flow, dense bands were seen and had curved trajectories (Figure 3).

### Blood Flow Velocity in Pathways with and without Dark Tail Flow at a Bifurcation

A total of 48 bifurcation points, where dark tail flow bisected into a dark tail and a dark tail-free flow, were analyzed. Additionally, a total of 96 dark tails and 48 plasma gaps were analyzed in 5 normal subjects to examine velocity differences in moving objects between the parent vessel and the 2 daughter vessels. The average velocity of dark tails was 1.30±0.27 mm/s (range, 0.59–2.11) in parent vessels and 1.12±0.25 mm/s (range, 0.61–1.98) in daughter vessels with dark tails (p = 0.003) (Table 1). When velocity measurements were averaged in both types of vessels, dark tail velocity was 1.21±0.26 mm/s. The average velocity of plasma gaps in daughter vessels without a dark tail was 0.64±0.11 mm/s (range, 0.26–1.13), which was significantly lower than dark tail velocity in both the parent (p<0.001) and daughter (p<0.001) vessels.

**Table 1:** Differences in average velocity, vessel diameter, and angle of bifurcation between the vessel before bifurcation, after bifurcation with dark tail flow, and after bifurcation without dark tail flow.

Subject	Average Velocity(mm/sec)				Average Diameter(µm)				Average Angle(degree)		
	Before bifurcation	After bifurcation with dark tail flow	After bifurcation without dark tail flow	P value	Before bifurcation	After bifurcation with dark tail flow	After bifurcation without dark tail flow	P value	After bifurcation with dark tail flow	After bifurcation without dark tail flow	Pvalue
A	1.34±0.39	0.91±0.10	0.69±0.11		7.7±0.98	8.9±2.2	8.3±1.2		45.8±21.7	87.8±24.9	
B	0.92±0.21	1.12±0.35	0.47±0.17		8.2±1.7	7.8±1.2	7.9±1.1		37.9±25.6	100.2±15.5	
C	1.67±0.19	1.52±0.43	0.75±0.15		8.6±1.1	10±1.3	9.1±2.4		34.0±12.4	81.1±38.8	
D	1.26±0.24	1.16±0.17	0.71±0.17		8.9±3.0	7.8±1.3	8.1±2.1		51.7±43.2	84.5±24.6	
E	1.32±0.37	0.91±0.26	0.58±0.19		8.8±0.96	8.8±1.3	9.6±2.7		48.8±31.6	81.0±27.5	
Average	1.30±0.27	1.12±0.25	0.64±0.11	P<0.0001	8.4±1.6	8.7±1.6	8.6±1.9	P = 0.88	43.6±26.5	86.9±25.3	P<0.001

### Bifurcation Vessel Diameter and Angle of Pathways with and without Dark Tail Flow

The average vessel diameters of parent vessels, daughter vessels with dark tail flow, and daughter vessels without dark tail flow were 8.4±1.6 µm, 8.7±1.6 µm, and 8.6±1.9 µm, respectively (Table 1). The average vessel diameter was not significantly different in parent vessels and in daughter vessels with (p = 1.0) or without (p = 1.0) dark tail flow. There was also no difference between daughter vessels with and without dark tail flow (p = 1.0). The average angle between parent and daughter vessels with dark tail flow was 43.6°, and the angle between parent and daughter vessels without dark tail flow was 86.9°(Table 1). The angle between parent and daughter vessels with a dark tail flow was significantly smaller than the angle between parent and daughter vessel without a dark tail flow (p<0.0001).

## Discussion

In this study, erythrocyte aggregates were observed as dark tails on AO-SLO images. The distribution of dark tails in the parafoveal capillary network and their behavior at vessel bifurcations were explored in normal subjects. The AO-SLO imaging revealed two different pathways in the parafoveal capillary network; pathways with dark tails and pathways without dark tails. This suggests that erythrocyte aggregates have preferential pathways through the retinal microcirculation. Moreover, daughter vessels with dark tail flow formed smaller angles with parent vessels at bifurcation points and had higher blood velocities than daughter vessels without dark tail flow. Therefore, bifurcation angle and blood flow velocity may influence which path erythrocyte aggregates prefer.

Previous studies have focused on blood flow characterization in the retinal microcirculation, in which preferential leukocyte pathways were documented. Nishiwaki et al. [12] identified retinal leukocyte “preferential channels” in the rat, in which leukocytes predominantly flowed. These channels were characterized by a high flow velocity and a straight, short capillary route. These preference prevented leukocytes from entering small capillaries, where they would have likely become stuck. Tam et al. [18] found leukocyte-preferred paths (LPPs) and plasma gap capillaries (PGCs) in the human retinal circulation using AO-SLO. They theorized that LPPs might prevent leukocytes from entering non-LPP capillaries and that PGCs may serve as relief valves when a leukocyte enters a nearby LPP.

Our results are in agreement with the Tam et al. study. [18] The dark tail-free pathway was very similar to their PGCs. The difference was that they focused on bright particles, identified as leukocytes, while we focused on dark regions, identified as erythrocyte aggregates. They measured leukocyte speeds in LPP segments as 1.80 mm/s, which were significantly higher than plasma gaps in PGC segments (1.30 mm/s), but not in LPP segments (1.73 mm/s). Although we did not analyze plasma gap velocity in dark tail pathways, which are smaller and likely heavily influenced by both erythrocyte aggregates and leukocytes, we did examine plasma gaps in dark tail-free pathways. The observed differences between reports may have been caused by inherent study differences. Firstly, their data was obtained from 1 healthy subject. Secondly, we measured plasma gap velocity only in vessels without dark tail flow. As Figure 3C shows, extremely slow velocities were included in analyses of vessels without dark tail flow.

Following bifurcation, blood flow velocity in daughter vessels with dark tail flow (range, 0.61–1.98 mm/s) was significantly higher than in daughter vessels without dark tail flow (range, 0.26–1.13 mm/s). In addition, velocities within vessels without dark tails were more variable. Figure 3C shows ST images of vessels without dark tail flow. The ST images consisted of both straight and curved lines, and the curved lines may represent periodical velocity change. The range of velocities measured from Figure 3C varied between 0.10 and 0.68 mm/s and included extremely slow blood flow in vessels without dark tail flow. One of the possible causes of these slow flow velocities was the influence of pulsation. As indicated in Figure 3, trajectories on the ST images showed a regular cycle of changing in slope, suggesting that pulsation changed the velocity of blood components periodically. All of these observations suggest that blood flow velocity evaluation in the parafoveal capillary network can account for velocity differences in vessels with and without dark tail flow, which should be analyzed separately.

The results showed great variability in the length of dark tail vessels among the subjects. One possible reason for this variability is the wide interindividual variability in the size of the FAZ. Because the areas of analysis were determined on the basis of the FAZ boundary in order to extract the area with the capillary monolayer, total extension of the vessels might be substantially influenced by FAZ size. On the other hand, dark tail flow distribution varied slightly between images recorded on separate days. As shown in Figure 4, even when different pathways existed, some commonality was present. These slight pathway changes were likely caused by flow frequency, not by absolute blood flow. Therefore, the current study showed that erythrocyte aggregates have preferential pathways and that this preference has minimal change.

The hemodynamics of erythrocyte aggregates could be a potential biomarker of microcirculatory disturbance in vascular diseases. Hemorheological disturbances (e.g., decreased erythrocyte deformability [33] and increased erythrocyte aggregation [31], [32]) are known to occur in diabetics and are thought to be associated with erythrocyte hyperaggregation, which would promote pathologic blood flow distribution in nutritive capillaries. [34] Erythrocyte aggregation also increases in patients with systemic lupus erythematosus (SLE), which could decrease blood flow and contribute to thromboembolitic processes in SLE patients. [35] Future investigations on erythrocyte aggregate flow frequency and distribution in parafoveal capillaries of diseased eyes are planned.

Because erythrocytes and leukocytes move in a single file and erythrocytes cannot overtake leukocytes in the capillary lumen, we assumed that the dark tail head velocity was approximately that of leukocytes. In support of this assumption, average dark tail velocity in retinal capillaries was close to that of leukocytes, as measured by Martin et al. [19], [28] with AO-SLO, who reported an average leukocyte velocity of 1.37 mm/s [28] and 1.30 mm/s. [19] However, values obtained in the current study were considerably lower than those that we obtained in a previous study (1.49 mm/s). [22] This difference may have been caused by differences in vessels examined and by variation between data selection. In the current study, we chose series of vessels, which consisted of 1 parent and 2 daughter vessels, but in our previous study, we chose vessels at random, regardless of bifurcation influence. Another explanation could be the influence of cardiac cycle on blood flow on velocity, as reported by Martin et al. [19] and Zhong et al. [27] Measurement of pulsatility would reveal cyclic changes in blood flow velocity, which would make calculations of mean velocity more accurate. We did not assess the pulsatility cardiac cycle in our previous report, but this was done in the current study.

Our study had several limitations due to the relatively low number of bifurcations analyzed and image resolution. Several different patterns of blood flow occurred at bifurcations, as observed on AO-SLO images, and only vessels consisting of a parent vessel and a daughter vessel with and without dark tails were chosen for analysis. This decreased the number of bifurcations meeting inclusion criteria to 48.

In conclusion, AO-SLO noninvasively revealed the existence of pathways with and without dark tail flow in normal subjects. Pathways with dark tail flow in daughter vessels had a faster blood flow and a smaller bifurcation angle than daughter vessels without dark tail flow. Therefore, AO-SLO is an informative tool for examining retinal microcirculatory hemodynamics.
